# Dysfunction of Emotion Regulation in Mild Cognitive Impairment Individuals Combined With Depressive Disorder: A Neural Mechanism Study

**DOI:** 10.3389/fnagi.2022.884741

**Published:** 2022-07-22

**Authors:** Meng Liu, Jing Ma, Chang-Yong Fu, Janelle Yeo, Sha-Sha Xiao, Wei-Xin Xiao, Ren-Ren Li, Wei Zhang, Zeng-Mai Xie, Ying-Jie Li, Yun-Xia Li

**Affiliations:** ^1^Department of Neurology, Tongji Hospital, School of Medicine, Tongji University, Shanghai, China; ^2^Faculty of Science, University of Sydney, Camperdown, NSW, Australia; ^3^School of Communication and Information Engineering, Shanghai University, Shanghai, China

**Keywords:** mild cognitive impairment, depressive symptoms, emotion regulation, neural oscillations, functional connectivity

## Abstract

Depression increases the risk of progression from mild cognitive impairment (MCI) to dementia, where impaired emotion regulation is a core symptom of depression. However, the neural mechanisms underlying the decreased emotion regulation in individuals with MCI combined with depressive symptoms are not precise. We assessed the behavioral performance by emotion regulation tasks and recorded event-related electroencephalography (EEG) signals related to emotion regulation tasks simultaneously. EEG analysis, including event-related potential (ERP), event-related spectral perturbation (ERSP), functional connectivity and graph theory, was used to compare the difference between MCI individuals and MCI depressed individuals in behavioral performance, the late positive potential (LPP) amplitudes, neural oscillations and brain networks during the processing of emotional stimuli. We found that MCI depressed individuals have negative preferences and are prone to allocate more attentional resources to negative stimuli. Results suggested that theta and alpha oscillations activity is increased, and gamma oscillations activity is decreased during negative stimulus processing in MCI depressed individuals, thus indicating that the decreased emotion regulation in MCI depressed individuals may be associated with enhanced low-frequency and decreased high-frequency oscillations activity. Functional connectivity analysis revealed a decrease in functional connectivity in the left cerebral hemisphere of the alpha band and an increase in functional connectivity in the right cerebral hemisphere of the alpha band in MCI depressed individuals. Graph theory analysis suggested that global network metrics, including clustering coefficients and disassortative, decreased, while nodal and modular network metrics regarding local nodal efficiency, degree centrality, and betweenness centrality were significantly increased in the frontal lobe and decreased in the parieto-occipital lobe, which was observed in the alpha band, further suggesting that abnormal alpha band network connectivity may be a potential marker of depressive symptoms. Correlational analyses showed that depressive symptoms were closely related to emotion regulation, power oscillations and functional connectivity. In conclusion, the dominant processing of negative stimuli, the increased low-frequency oscillations activity and decreased high-frequency activity, so as the decrease in top-down information processing in the frontal parieto-occipital lobe, results in the abnormality of alpha-band network connectivity. It is suggested that these factors, in turn, contribute to the declined ability of MCI depressed individuals in emotion regulation.

## Introduction

Mild cognitive impairment (MCI) is a state between the expected cognitive decline of normal aging and the more severe progression of dementia, and the prevalence of depression is higher in MCI individuals than in younger and normal elderly populations (Copeland et al., 2004; Lee and Shinkai, 2005). Studies have shown an increased risk of MCI combined with depression progressing to dementia (Diniz et al., 2013). Depression is characterized by emotional dysfunction (Carl et al., 2018), and emotion regulation is also a major cognitive ability in humans. Altered emotional responses and associated emotion dysregulation have been found to play an important role in depression and related psychological disorders (Bylsma et al., 2008). Decreased emotion regulation in MCI individuals may be an important reason they are prone to depressive symptoms. Depression is a common comorbid condition in MCI stages (Ismail et al., 2017). However, most studies have focused on the prognostic role of affective disorders in MCI, and the mechanisms of impaired emotion regulation processing in individuals with MCI combined with depressive symptoms have not been well clarified. We want to explore the relationship between depression with cognition and emotion regulation, and how depressive symptom effect the emotion preprocessing and its potential mechanism. Neuropsychological tests are widely used for emotion state assessment. However, they are highly influenced by subjective factors, and some individuals may conceal or may have a distinct definition of the situation they experience, so there is a need to develop an objective and accurate detection method. With the development of electrophysiology, event-related potential (ERP) is widely used to examine brain evoked potential induced by multiple stimuli to reflect the physiological changes of brain nerves during the respective cognitive process, also known as cognitive potential (Zhong et al., 2019). ERP is obtained from scalp electroencephalography (EEG) and is favored by many researchers because of its affordability, non-invasiveness, and safety. Preliminary evidence suggested that late positive potential (LPP) may serve as an indicator of a sustained increase in neural activity following the presence of emotional stimuli. Most tests of emotion regulation of LPP are based on the magnitude of LPP amplitude, which reflects the ability to use emotion regulation for cognitive reappraisal (Kennedy and Montreuil, 2020). Some studies have found a significant increase in LPP amplitudes in depressed individuals for negative stimuli (Dainer-Best et al., 2017). However, some studies have come to the conclusion that the LPP response was not associated with concurrent depressive symptoms, suggesting the LPP is a relatively stable and reliable measure of emotional processing. The conflicting results might be partly explained by differences in the diagnostic criteria of the study groups or sample size. We believe that to clarify the relationship between depression and LPP. It may be necessary to study different larger samples and more consistent depression scores (McGhie et al., 2021).

Patients with MCI have severe working memory impairment. Theta oscillation plays a crucial role in working memory, especially the loss of theta oscillation in the frontal region may be one of the essential mechanisms of working memory impairment (Pomper and Ansorge, 2021). The oscillations activity of EEG rhythms has a vital role in processing emotional stimuli and is susceptible to emotional intensity and potency (Lewis, 2005; Knyazev, 2007; Bekkedal et al., 2011). The activity of theta rhythms is more sensitive to emotional stimuli, and negative pictures with higher arousal enhance the activity of theta rhythms (Aftanas et al., 2001; Balconi and Lucchiari, 2006; Balconi and Pozzoli, 2009). Oscillations of Beta are associated with top-down control of behavior (Stoll et al., 2016), while studies have also confirmed the relevance of emotion regulation to cognitive control (Moser et al., 2010; Ochsner et al., 2012). For neutral stimuli, emotional stimuli can induce stronger gamma oscillation (Müller et al., 2000; Balconi and Lucchiari, 2008). Oscillations of gamma are also correlated with some cognitive processes involved in emotion regulation, such as attention and memory (Kaiser and Lutzenberger, 2003). In a study by Muller, greater gamma power was induced during selective attention stimulation (Müller et al., 2000). The intensity of Alpha wave activity is inversely proportional to the intensity of the corresponding cortical activity, where a more robust alpha wave activity indicates a weaker brain activity (Kline et al., 2007). Several studies have focused on the changes in power oscillations in individuals with MCI or depression. The EEG rhythms of MCI patients have been found to differ from those of healthy older adults. Studies have also shown increased theta wave power in MCI patients compared to healthy controls (Yu et al., 2021). In addition, MCI patients with higher alpha amplitude show greater cortical atrophy (Moretti, 2015). Our previous study found that, compared with MCI-failure group, the MCI-Success group demonstrated larger theta power to negative images. However, the healthy elderly controls success group demonstrated stronger theta power to images preceded by more neutral descriptions compared with the healthy elderly controls -failure group (Xiao et al., 2021). While subjects in major depression performing emotion-related tasks show decreased frontal cortex gamma power (Lee et al., 2010; Liu et al., 2014). However, the characteristics of power oscillations in the processing of emotional stimuli in combined MCI and depressed individuals are unclear. The effect of depression on MCI is unclear and needs further research.

Functional connections affect emotional processing. Many articles focus on brain connectivity in MCI patients with functional magnetic resonance imaging (fMRI) techniques (Sun et al., 2020). However, in recent years, EEG has had some advantages over fMRI in studying functional connectivity (Mele et al., 2019). The assessment of functional connectivity (FC) and network topology can provide an integrative approach to reflect progressive brain dysfunction in MCI and Alzheimer’s disease (AD) (Hallett et al., 2020). The abnormal FC is underlying the decreased emotion regulation in individuals with MCI. Many studies have also found that there are apparent functional connectivity abnormalities in depressed people. However, there are few studies on how depressive symptoms affect the MCI population, which needs further research.

Dysfunction and dysregulation of emotion-related brain circuits may contribute to emotion disorders (Phillips, 2003). Studying the neural activity of emotional stimuli is critical to understanding the emotional information processing in the brain. It may also provide clues to how emotions can be regulated in the treatment of neuropsychiatric disorders in the future. Enhanced knowledge of the neural mechanisms underlying emotion regulation could help improve understanding of the complex interplay of emotion and cognition underlying human behavior. However, the underlying neural mechanisms of how depressive symptoms affect MCI individuals’ ability to regulate, how depressive symptoms affect emotion regulation loops, and what relationship exists between depressive symptoms and emotion regulation and cognitive function have not been clarified. Therefore, in this study, we propose to recruit MCI individuals and compare the differences in emotion regulation ability between MCI and combined MCI and depressed individuals to investigate the underlying neural mechanism in the impairment of the emotion regulation loop by observing the changes in the amplitude of ERP component LPP, power oscillations in each frequency band, as well as the changes happening in the brain functional connectivity. We attempt to lay a solid foundation in understanding the process of the emotion regulation loop to devise necessary intervention mechanisms to improve the quality of life of these individuals.

## Materials and Methods

### Participants

Forty-three subjects who met the diagnostic criteria of MCI were included in the outpatient clinic of the Department of Neurology of Tongji Hospital for memory loss. A simple power calculation for a two-sided *t*-test with *d* = 0.8 (i.e., large effect), alpha = 0.05 and *n* = 26 yields a power of 80%. However, measuring EEG in more subjects for large effects is not very common in the EEG literature. Since unwanted noise usually influences EEG recordings, a small effect size is understandable. Subjects with a total Hamilton Depression Rating Scale (HAM-D) scores (≥14) and total depression core symptom score (≥3) were included in the MCI combined depressive symptoms group (*n* = 17). Subjects with a total HAMD depression score (<7) and total depression core symptom score (=0) were included in the MCI group (*n* = 26) and were matched for age, gender, education level, and cognitive domain scores on the set of neuropsychological tests. The same physician conducted a psychological medical evaluation of all subjects. Independent psychological medical evaluations are performed by physicians who have no relationship with the examined individual. The study was approved by the Ethics Committee of Shanghai Tongji Hospital [(Tong) Audit No. (K-2017-003-xz–90130)], and all subjects signed an informed consent form.

#### Mild Cognitive Impairment Enrollment Criteria

(1) Complaints of cognitive decline or cognitive decline reported by an informed person.
(2) The Mini-Mental State Exam (MMSE) scores: illiterate ≤ 17, elementary school ≤ 20, middle school and above ≤ 24; or Montreal Cognitive Assessment Basic (MoCA-B) scores: elementary school and below ≤ 19, secondary school ≤ 22, college ≤ 24.
(3) Clinical Dementia Rating Scale.
(CDR) = 0.5; not at the level of AD dementia.
(4) Meeting any one of the following three criteria: (1) impairment of 2 metrics in the same cognitive domain (>1 SD); (2) impairment of 1 test score in 2 or more of the four cognitive domains (>1 SD); (3) instrumental activities of daily living (IADL) score: more than one item score of 1 or more.

Depressive symptoms were defined according to the Diagnostic and Statistical Manual of Mental Disorders (DSM-V). The severity of depressive symptoms was scored using the 17-item Hamilton Depression Scale (HAMD-17); a total HAMD-17 score ≥ 14 and a core depressive symptom score ≥ 3 were considered to have depressive symptoms; a total HAMD-17 score < 7 and a core depressive symptom score ≤ 0 were considered to have no depressive symptoms.

#### Exclusion Criteria

(1) Those who had severe visual, hearing, aphasia and other physical diseases could not complete the neuropsychological tests.
(2) History of other definite neurological diseases (e.g., Alzheimer’s disease, Vascular dementia, Parkinson’s disease, Huntington’s disease, Hydrocephalus, Epilepsy, Brain injury, etc.).
(3) Have a clear history or evidence of other internal diseases, such as diabetes, thyroid disease, vitamin B12 deficiency, substance abuse, nutritional, metabolic diseases, etc.

### Demographic Data Assessment

All individuals were required to sign an informed consent form and complete relevant tests {cranial CT or MRI scan, biochemical blood tests such as folic acid, vitamin B12, thyroid function [free triiodothyronine (FT3), free tetraiodothyronine (FT4), thyroid-stimulating hormone (TSH)], syphilis spirochete and HIV antibody tests} before enrollment. All subjects were required to complete a battery of neuropsychological tests, including general cognitive functioning assessment (MMSE and MoCA-B), memory assessment [Hopkins Verbal Learning Test (HVLT), Wechsler logical memory], executive functioning assessment [The Shape Trail Test (STT)], language functioning assessment [Boston Naming Test (BNT) and Verbal Fluency Test (VFT)], and visuospatial functioning assessment the Rey–Osterrieth complex figure test (ROCF). Depressive symptoms were assessed by HAMD-17, which has good reliability and validity. The HAMD- 17 items scale includes depressed emotion, behavioral agitation, social withdrawal, despair, and lack of energy. All subjects’ neuropsychological tests were completed by the same experienced neuropsychological evaluator.

All subjects were asked to complete a set of neuropsychological assessments and depressive symptoms during the screening phase in a quiet and undisturbed environment where subjects remained relaxed.

### Stimuli and Procedure

A total of 90 pictures (60 negative and 30 neutral) were selected from the International Affective Picture System (IAPS) (Lang et al., 1997), size 416 × 312.75 of the 90 images were identical to Hajcak and Foti (2008). The number of negative, neutral, and re-appraisal picture for each block are balanced. Valence ranged from 1 (most negative) to 9 (most positive), and arousal ranged from 1 (calm) to 9 (highly aroused). The participants were told that they would be viewing faces depicting a range of emotions. All the participants performed practice trials, the experiment did not begin until the subjects understood the task, and the practice trials were not included in the experiment task. Prior to each picture, a short description of the upcoming picture was displayed on the screen and then broadcasted through normal intonation and speed. The experiment consisted of three situations: neutral viewing, harmful viewing, and negative reappraisal. The researchers used 30 neutral pictures in the neutral viewing case, preceded by a neutral description. A negative description was previously added in half of the 60 unpleasant pictures. The other half was described with more neutral or positive word descriptions. In all conditions, all subjects were asked to read the descriptions and then take the word descriptions to understand the pictures and rate the valence and arousal according to their feelings. In the adverse reappraisal condition, these descriptions served as a guide for subjects to perform cognitive reappraisal. The images were presented sequentially on a 15.6-inch LCD screen at a viewing distance of approximately 70 cm using E-Prime 3.0 stimulus presentation software (Psychology Software Tools, Inc., Pittsburgh, PA, United States). All tests were conducted in a quiet room.

**FIGURE 1:**
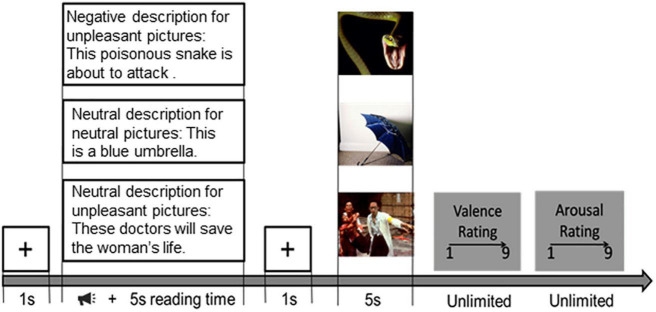
Emotion regulation task procedure. Then each picture would be preceded by a description of the upcoming picture. When the sound ended, the subject would read the description and have more than 5 s to read it for the subject to understand its meaning. After the reading, a black fixation cross appears on a black screen for 1 s. During the presentation of the stimuli, participants were asked to understand the picture based on the preceding textual description. After each picture was presented, participants were required to give their rating scores (1 = negative, 9 = positive) and arousal (1 = calm, 9 = aroused) of each picture.

The paradigm described in Hajcak and Foti (2008) was employed in this study, and the procedure is shown in Figure 1. After a brief introduction to the experiment, three training trials were conducted to ensure the participant’s understanding of the procedure. During the introduction, participants were informed that they would be viewing pictures; each picture would be preceded by a description of the upcoming picture. When the sound ended, the subject would read the description and have more than 5 s to read it for the subject to understand its meaning. After the reading, a white gaze cross appears on a black screen for 1 s, telling the subjects that the reading time is over and drawing their attention to the center of the screen. During the presentation of the stimuli, participants were asked to understand the picture based on the preceding textual description. After each picture was viewed, participants were asked to rate the reaction (1 = negative, 9 = positive) and arousal (1 = calm, 9 = aroused) of each picture using a self-assessment model (Bradley and Lang, 1994). The researcher would explain the meaning of these two-dimensional rating scales in detail to each participant. Participants were told to choose a score from these two scales that best represented their feelings about the previous picture. In the actual experiment, the subjects’ understanding was verified by the researchers, including the subjects’ understanding of the sequence of events and their understanding of the meaning of valence and arousal. The emotion regulation task consists of 5 blocks. Each included 18 trials. The trial order for each subject was determined randomly. There were three types of tasks in each block: neutral viewing, harmful viewing, and negative reappraisal. While the neutral viewing trials served as a baseline, the negative viewing trials were used to elicit uncontrolled emotional responses to negative images. The purpose of the negative-reappraisal trials was to capture the cognitive reappraisal process performed by the participants (Figure 1).

### Electroencephalography Recording and Data Preprocessing

Electroencephalography recordings were made using the Neuroscan system (Neuroscan SynAmps2, Compumedics, United States, INC). Based on the 10–20 system, 64 scalp electrodes were recorded, and two electrodes were placed on the right and left mastoids. Blinks and eye movements were recorded at four facial electrodes: the upper and lower two approximately 1 cm to the left eye, one approximately 1 cm to the left eye, the left one, and the right one approximately 1 cm to the right eye. The data preprocessing and analysis were performed in MATLAB software using the EEGLAB toolbox (Delorme and Makeig, 2004). The EEG sampling frequency was 1,000 Hz. All data were re-referenced to the average of two mastoids and band-pass filters of 0.1 and 95 Hz. The EEG was segmented for each trial, starting 200 ms before the appearance of each image and continuing for 5,000 ms. ERPs were averaged from trials for each of the three conditions: neutral pictures followed by a neutral description, unpleasant pictures followed by a negative description, and unpleasant pictures followed by a more neutral description. The average activity within the 200-ms window before the image appeared was used as the baseline for each ERP average. EEG data preprocessing to ensure that each subject had at least 15 trials per condition (i.e., subjects who could retain 50% of the trials were included in the study).

### Event-Related Potential Analysis

We first performed an exploratory analysis of LPP (Sabbagh and Taylor, 2000; Flaisch and Schupp, 2013; Zanon et al., 2018), thus identifying groups of adjacent electrodes that showed different conditions of LPP. According to the results of exploratory analysis of LPP and referred to the previous study (Guevarra et al., 2020), we divided the LPP time window into five parts, including win1 (400–1,000 ms), win2 (1,000–2,000 ms), win3 (2,000–3,000 ms), win4 (3,000–4,000 ms), win5 (4,000–5,000 ms). We used a one-way ANOVA to compare LPP amplitudes in MCI, and MCI combined depressive symptom groups in response to different types of emotional stimuli (negative, neutral, and reappraisal). In five windows after stimulus onset, 400–1,000 ms (early window), 1,000–2,000 ms (middle window), 2,000–3,000 ms (late window), 3,000–4,000 ms (late window), 4,000–5,000 ms (late window), for each window, LPP was quantified as the in-window moderate activity (Xiao et al., 2021; Ding et al., 2022). The ERP analysis was conducted using ERPLAB toolbox as well as related MATLAB code.

### Event-Related Spectral Perturbations Analysis

We computed ERSP values during the emotion regulation task to assess brain dynamics in time-frequency domains. The ERSP analysis was performed by short-time Fourier transform (STFT) to compute the baseline spectrum from the EEG prior to each event. The amplitude spectra of these windows were moving averaged by dividing short, overlapping data windows. The ERSP was computed by subtracting the spectral transform of each response from the respective average baseline spectrum. The ERSPs for many trials were then averaged to produce an average ERSP plotted in the time-frequency plane as the relative spectral log amplitudes. ERPs were computed using a moving Hanning window (200 ms) wavelet with a minimum frequency of 1 Hz, and the highest frequency was analyzed (40 Hz) in steps of 0.5 Hz. ERSP analysis was performed from the start of the stimulus sequence to the presentation of the exploration sequence within 5,000 ms. The baseline time was 1,000 ms before the presentation of the stimulus sequence. The mean ERSP was computed separately for each subject and each electrode. For all brain regions, the difference in ERSP values across frequency bands (theta, alpha, beta, and gamma) was computed for individuals with and without depression in MCI at all time points. The event-related desynchronization (ERD)/event-related synchronization (ERS) values were computed by averaging the absolute power obtained from the ERSP analysis over different frequency band ranges. The resulting ERD/ERS plots are plotted from −1 ∼5 s. The baseline time is 1,000 ms. The frequency bands of interest were theta (4–7 Hz), alpha (8–12 Hz), beta (13–30), and gamma (30–40 Hz) bands.

### Functional Connectivity Analysis

The phase-based approach’s weighted Phase Lag Index (wPLI) depends on the distribution of phase angle differences (Cohen, 2013). This phase will be more synchronized when the two channels are more functionally coupled. When both electrodes measure electrical activity from the same source, a phase lag from zero to π is generated. Weighted phase lag is a method to avoid volume conduction due to zero phase lag connectivity (Cohen, 2013). It measures whether the phase angle difference distribution is toward the complex plane’s positive or negative side (Cohen, 2013). The more powerful the phase angle difference on the same side, either positive or negative, the higher the wPLI. wPLI connectivity is computed for each electrode pair providing a value between 0 and 1 at each frequency and time point. Higher values indicate more phase synchronization between the two electrodes (Vinck et al., 2011). The equation for calculating wPLI is as follows:


(1) $$W\,P\,L\,I_{x\,y}=\frac{n^{-1}\,\sum_{t=1}^{n}\left|I\,m\,a\,g\,\left(s_{x\,y\,t}\right)\right|\,s\,g\,n\,\left(i\,m\,a\,g\,\left(s_{x\,y\,t}\right)\right)}{n^{-1}\,\sum_{t=1}^{n}\left|i\,m\,a\,g\,\left(S_{x\,y\,t}\right)\right|}$$

Where imag(xyt) denotes the mutual spectral density at time point t in the complex plane XY. Sgn is the sign: (−1, +1, or 0). We extracted the wPLI values of the θ wave (4–7 Hz), α wave (8–13 Hz), β wave (14–30 Hz), and γ wave (30–40 Hz) in the frequency bands of 3–5 s within the time window of LPP occurrence, respectively. For significant main effects or interactions, follow-up tests were FDR corrected for multiple comparisons.

### Graph Theory Analysis

To obtain the graph-theoretical metrics of brain regions related to emotion regulation, GRETNA (graph theoretical network analysis) software was used in MATLAB for theoretical graph analysis (Wang et al., 2015). After obtaining the 60 × 60 functional connectivity matrixes by weighted phase lag synchronization, the threshold was applied from the correlation matrix. The adjacency matrix was computed, and the binary undirected graph was obtained. Since different thresholds generate graphs with different connection densities or sparsity, it is generally recommended to calculate network properties in a wide threshold range. The threshold range chosen for this study was 0.15–0.85 (Langer et al., 2012; Arnold et al., 2014). Global network metrics including small-world efficiency, hierarchy, synchronization and assortative nodal and modular network metrics include efficiency, local efficiency, shortest path, degree centrality and clustering coefficient. For significant main effects or interactions, follow-up tests were FDR corrected for multiple comparisons.

### Statistical Analysis

SPSS (Version 20.0, Tongji University, China) was used for statistical analysis. Data were expressed as mean ± standard deviation values or percentages, and demographic information as described. Emotion regulation perceived behavioral performance and other related cognitive function test results, including Hopkins word learning, Boston naming, word fluency, STTl, and Rey-O complex graphics test scores, were statistically evaluated using independent samples *t*-tests. Behavioral performance findings, including arousal rating scores and valence scores, were statistically evaluated using independent samples *t*-tests, and pair-*t*-tests were applied to compare the difference between negative stimulus pictures and reappraisal stimulus pictures. Electrophysiological results, including amplitude and spectral power of the ERP component LPP related to emotion regulation tasks and functional connectivity index wPLI values, were compared using independent *t*-tests. Bilateral *p* < 0.05 was considered statistically significant.

## Results

### Demographic Information

The subjects were matched for age, gender and education, and other demographic factors did not show any significant differences. Since the subjects included in our study were the MCI population, grouped according to the presence or absence of depressive status, there were significant differences in HAMD scores between these two groups (Table 1).

**TABLE 1:** Demographic data.

	MCI group (*n* = 26)	MCI depression group (*n* = 17)	*p*-value
Age	64 ± 12.49	58.24 ± 15.79	0.22
Gender (female/male)	16/10	11/6	0.83
Education years	10.24 ± 4.24	8.35 ± 4.89	0.20
MMSE scores	25.19 ± 2.56	23.65 ± 4.69	0.17
Immediate memory	14.85 ± 5.31	16.22 ± 5.13	0.4
Delayed recall (5 min)	3.39 ± 3.13	4.0 ± 2.52	0.5
Delayed recall (20 min)	3.42 ± 3.0	3.77 ± 2.63	0.71
Logical memory	6.50 ± 3.18	5.41 ± 4.72	0.25
Boston naming	19.39 ± 4.15	17.24 ± 5.11	0.14
Verbal fluency	12.35 ± 4.61	9.59 ± 4.68	0.064
STT-A	73.35 ± 29.12	72.80 ± 32.40	0.96
STT-B	183.42 ± 67.41	157.87 ± 57.21	0.23
Rey-O (copy)	4.15 ± 1.52	4.12 ± 2.32	0.95
Rey-O (recall)	2.76 ± 1.76	3.12 ± 4.68	0.19
HAMD (score)	2.92 ± 1.76	17.29 ± 3.93	0.0001

*MMSE, Mini-Mental State Examination.*

### Behavioral Performance

**FIGURE 2:**
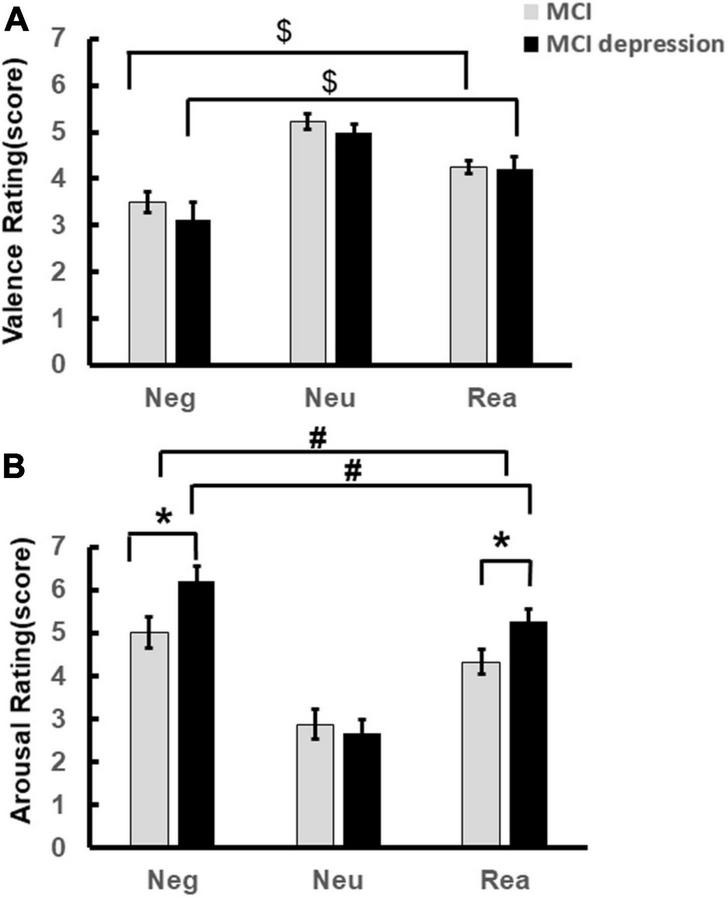
Emotion Regulation task behavioral performance. **(A)** Demonstrates the valence scores difference during the Emotion Regulation task regarding negative, neutral reappraisal stimuli. **(B)** Demonstrates the arousal scores difference during the Emotion Regulation task regarding negative, neutral reappraisal stimuli. *p < 0.05, MCI vs. MCI depression (regarding arousal to negative and reappraisal stimuli); ^#^*p* < 0.05, negative vs. reappraisal (regarding to the arousal scores); ^$^*p* < 0.05, negative vs. reappraisal (regarding to the valence scores).

The significant difference of arousal scores was illustrated in Figure 2A. Individuals with MCI comorbid depressive symptoms showed significantly stronger arousal regarding negative and reappraisal stimulus pictures. (Negative stimuli: 6.20 ± 1.49 vs. 5.11 ± 1.84, *p* = 0.048, *t* = −2.039, *df* = 46; Reappraisal stimuli: 5.27 ± 1.17 vs. 4.39 ± 1.46, *p* = 0.043, *t* = −2.084, *df* = 41). Significant condition effects for arousal and valence scores were found in each group, i.e., arousal to negative stimuli decreased with the application of reappraisal strategy in both groups (MCI comorbid depressive symptoms group: 5.27 ± 1.17 vs. 6.20 ± 1.49, *p* = 0.01, *t* = 2.904, *df* = 16; MCI group: 4.39 ± 1.46 vs. 5.11 ± 1.84, *p* = 0.021, *t* = 2.46, *df* = 25) (Figure 2A), whereas valence to negative stimuli was increased [(MCI depression group: 4.21 ± 1.11 vs. 3.11 ± 1.59, *p* = 0.017, *t* = −2.664, *df* = 16); MCI group: 4.25 ± 0.71 vs. 3.45 ± 1.13, *p* < 0.001, *t* = −4.258, *df* = 25] (Figure 2B); however, there was no significant difference in valence rating scores between groups (3.11 ± 1.59 vs. 3.45 ± 1.13, *p* = 0.42, *F* = 0.655, *df* = 1) (Figure 2B).

### Event-Related Potential Results

**FIGURE 3:**
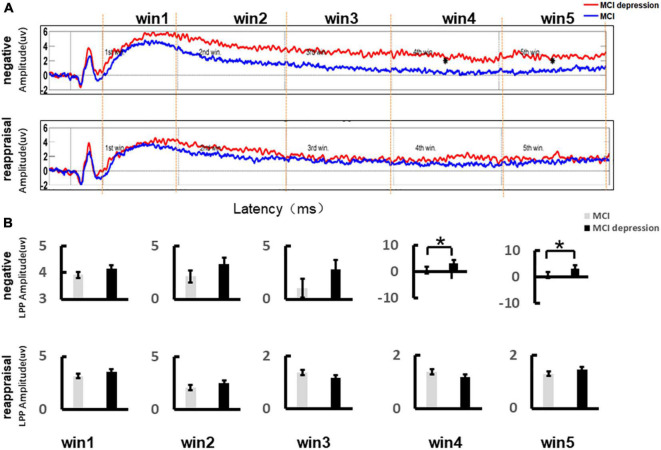
Grand average event-related potentials and amplitude differences of the LPP during the Emotion Regulation task. **(A)** Illustrates the event-related potential (ERP) amplitude in the Emotion Regulation trial; the LPP time window (Win3: 2,000–3,000 ms; Win4: 3,000–4,000 ms; Win5: 4,000–5,000 ms). **(B)** Demonstrates the LPP amplitude difference during the Emotion Regulation task between the MCI and MCI depression group. *p < 0.05.

According to previous studies (Qi et al., 2016), central areas are the most likely to have the largest LPP amplitudes, so we extracted the LPP amplitudes of the average of CZ, C2, and CPZ. Figure 3A illustrates the event-related potential (ERP) amplitude in the Emotion Regulation trial In the two time windows of win4 (3,000–4,000 ms) and win5 (4,000–5,000 ms), the LPP amplitude values were significantly higher in the MCI combined with depressive symptoms group compared to the controls group for negative stimulus pictures (win4: 3.06 ± 0.92 vs. 0.49 ± 0.51, *p* = 0.01, *t* = −2.654, *df* = 41; win5: 3.13 ± 0.92 vs. 0.69 ± 0.49, *p* = 0.015, *t* = 2.544, *df* = 41), while there no significant difference could be found in other time windows (Figure 3B).

### Event-Related Spectral Perturbation Results

**FIGURE 4:**
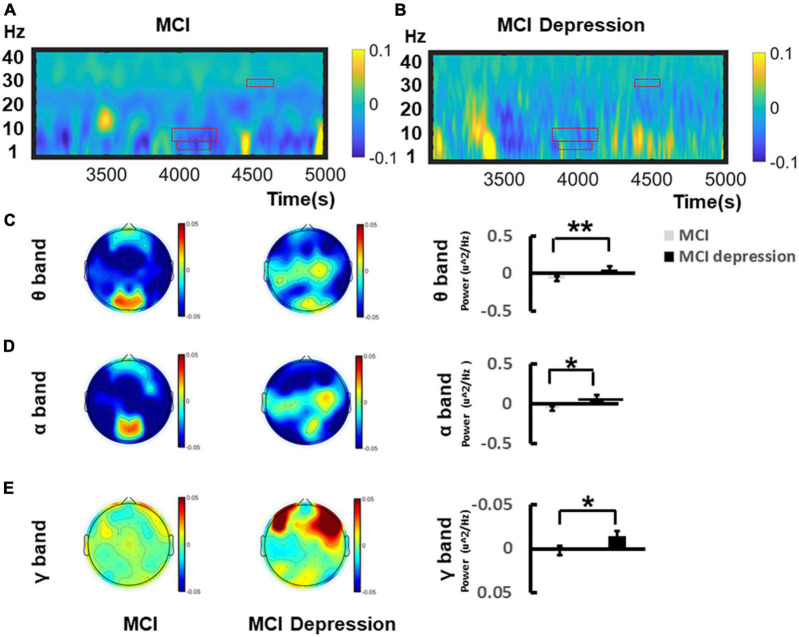
Grand average time–frequency distribution (TFD) over the central lobes during the time window of LPP of Emotion Regulation task. **(A)** Demonstrates the TFD over the central lobe (CP1/2, CPZ) ranging from (1–40 Hz) during the Emotion Regulation task regarding MCI group. The ROI of TFD were highlight with red frame. **(B)** Demonstrates the TFD over the central lobe (CP1/2, CPZ) ranging from (1–40 Hz) during the Emotion Regulation task regarding MCI depression group. **(C)** Illustrates the bar graph differences of ROI of theta oscillation between MCI and MCI depression group. **(D)** Illustrates the bar graph differences of ROI of alpha oscillation between MCI and MCI depression group. **(E)** Illustrates the bar graph differences of ROI of gamma oscillation between MCI and MCI depression group. *p < 0.05, ^**^p < 0.01.

The time-frequency distributions over the central lobe (CP1/2, CPZ) ranging from (1–40 Hz) during the Emotion Regulation task were demonstrated in Figures 4A,B. In the viewing of negative stimuli, during the late middle phase of the LPP (4,167–4,257 ms), MCI combined with depression individuals had significantly higher (0.057 ± 0.175 vs. −0.07 ± 0.15, *p* = 0.015, *t* = −2.545, *df* = 41) theta oscillations in the central electrodes (CZ, C2, and CPZ) compared to the controls group. During the late middle phase of the LPP (4,153–4,264 ms) (Figure 4C), in the mid-late stage of LPP (4,582–4,601 ms), MCI combined with depression individuals had significantly higher alpha oscillations in the central zone (CZ, C2, and CPZ) compared to controls (0.066 ± 0.16 vs. −0.065 ± 0.14, *p* = 0.008, *t* = −2.804, *df* = 41) (Figure 4D); in the mid-late stage of LPP (4,582–4,601 ms), MCI combined with depression individuals had significantly higher gamma oscillations was significantly decreased compared to controls (−0.015 ± 0.02 vs. 0.002 ± 0.03, *p* = 0.045, *t* = 2.073, *df* = 41) (Figure 4E) whereas there no significant difference can be found regarding beta (14–30 Hz) oscillations in the central areas (CZ, C2, and CPZ) compared to controls during the late middle phase of the LPP (4,161–4,207 ms) (0.02 ± 0.05 vs. 0.247 ± 0.07, *p* = 0.82, *t* = −0.235, *df* = 41).

### Functional Connectivity Characteristics

**FIGURE 5:**
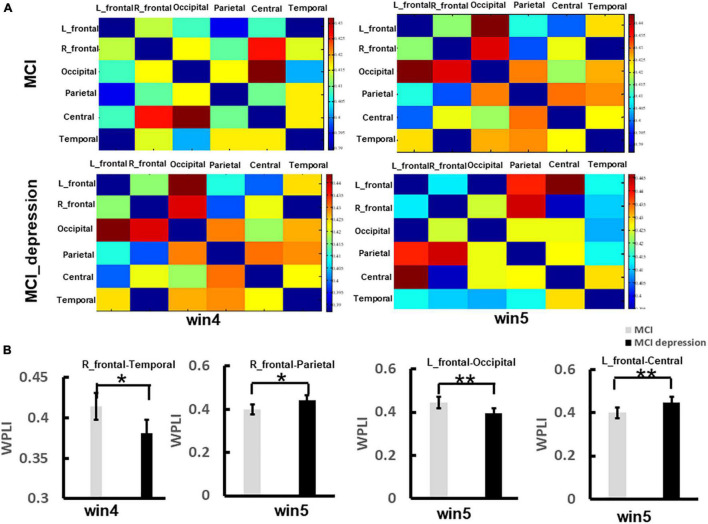
The significant difference of functional connectivity matrix between two groups during the Emotion Regulation task. **(A)** Illustrates the matrix of functional connectivity between 6 brain areas (left-frontal lobe, right frontal lobe, parietal lobe, occipital lobe, temporal lobe, and central regions) of two group. **(B)** Illustrates the group difference of right frontal-temporal lobe, right frontal-parietal lobe, left frontal-occipital lobe, left frontal-centrals lobe. *p < 0.05, ^**^p < 0.01.

The functional connectivity analysis was employed in the current study. We focused on brain regions, including the left frontal lobe, right frontal lobe, central areas, parietal lobe, occipital lobe, and temporal lobe. The matrix of functional connectivity between 6 brain areas (left-frontal lobe, right frontal lobe, parietal lobe, occipital lobe, temporal lobe, and central regions) of two group was shown in Figure 5A. In the time window (win4, 3,000–4,000 ms), the MCI combined with depressive symptoms. Patients present significantly lower functional connectivity between right frontal and temporal lobes than controls in response to negative stimulus (0.381 ± 0.04 vs. 0.414 ± 0.04, *p* = 0.013, *t* = 2.59, *df* = 41), which was observed in alpha band network; In time window (win5, 4,000–5,000 ms), the functional connectivity between left frontal and occipital lobes in patients with MCI combined with depression was significantly lower than controls in response to negative stimulus (0.393 ± 0.05 vs. 0.444 ± 0.05, *p* = 0.001, *t* = 3.461, *df* = 41). The functional connectivity between the left frontal lobe and central areas in patients with MCI combined with depressive symptoms was significantly higher than that in the control group in response to negative stimulus (0.448 ± 0.05 vs. 0.399 ± 0.06, *p* = 0.006, *t* = −2.89, *df* = 41), The functional connectivity between right frontal and parietal lobes in patients with MCI combined with depression was significantly higher than that in the control group (0.443 ± 0.05 vs. 0.398 ± 0.05, *p* = 0.005, *t* = −3.00, *df* = 41). However, in other frequency bands(θ, β, and γ), no significant difference can be found between groups (Figure 5B).

### Characteristics of Graph Theory

**FIGURE 6:**
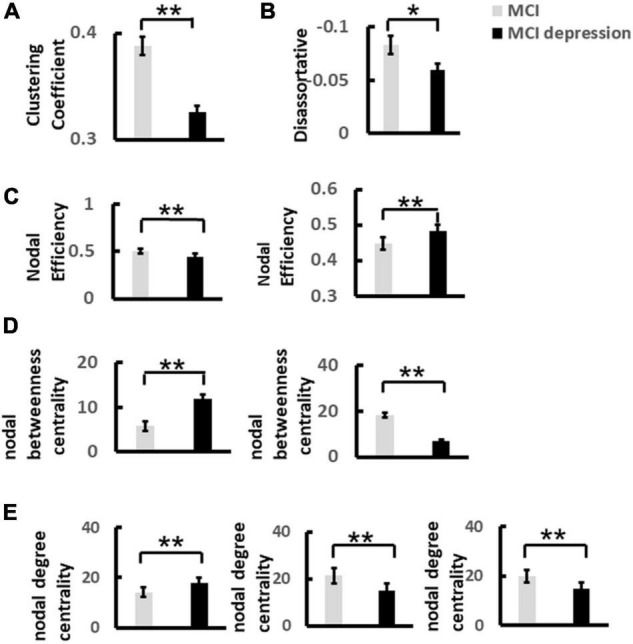
The significant difference of global and nodal network metrics for each group during the Emotion Regulation task. **(A)** Demonstrates the significant difference of Clustering Coefficient of alpha band over frontal lobe between MCI and MCI depression group. **(B)** Illustrates the significant difference of Disassortative of alpha band over frontal and parietal lobes between MCI and MCI depression group. **(C)** Illustrates the significant difference of Nodal efficiency of alpha band between MCI and MCI depression group. **(D)** Illustrates the significant difference of Nodal degree centrality of alpha band over frontal, parietal and occipital lobes between MCI and MCI depression group. **(E)** Demonstrates the significant difference of nodal betweenness centrality of alpha band over frontal, parietal and occipital lobes between MCI and MCI depression group *p < 0.05, **p < 0.01.

Based on the construction of 60 × 60 matrix as shown above, the results showed that there were significant differences in global network metrics including disassortative between the two groups, that is, the disassortative of patients with MCI combined with depressive symptoms were significantly lower than those of the control group (disassortative: −0.06 ± 0.03 vs. −0.08 ± 0.04, *p* = 0.05, *t* = −2.021, *df* = 41) (Figures 6A,B). Local network metrics including clustering coefficient showed significantly decreased compared with controls: (0.33 ± 0.02 vs. 0.39 ± 0.044, *p* < 0.0001, *t* = 5.359, *df* = 41). Other local network metrics such as nodal efficiency, was significantly higher in the frontal lobe than in the control group, while significantly lower in the parietal lobe (frontal lobe: 0.48 ± 0.03 vs. 0.45 ± 0.04, *p* = 0.003, *t* = 3.188, *df* = 41; parietal lobe: 0.44 ± 0.048 vs. 0.5 ± 0.03, *p* < 0.0001, *t* = 4.604, *df* = 41). The degree centrality in frontal lobe was significantly higher than that in control group, while in parietal lobe and occipital lobe was significantly lower than that in control group (frontal lobe: 18.0 ± 4.68 vs. 14.11 ± 4.02, *p* = 0.006, *t* = −2.90, *df* = 41; parietal lobe: 15.10 ± 5.20 vs. 21.36 ± 3.70, *p* < 0.0001, *t* = 4.613, *df* = 41; occipital lobe: 14.67 ± 3.90 vs. 19.74 ± 3.39, *p* < 0.0001, *t* = 4.51, *df* = 41). The betweenness centrality in frontal lobe was significantly higher than that in control group, while in parietal lobe was significantly lower than that in control group (frontal lobe: 10.44 ± 5.94 vs. 5.34 ± 4.10, *p* = 0.002, *t* = −3.34, *df* = 411; parietal lobe: 6.80 ± 6.0 vs. 18.28 ± 10.69, *p* < 0.0001, *t* = 4.019, *df* = 41) (Figures 6C–E).

### Correlation Between Depression and Emotion Regulation

**FIGURE 7:**
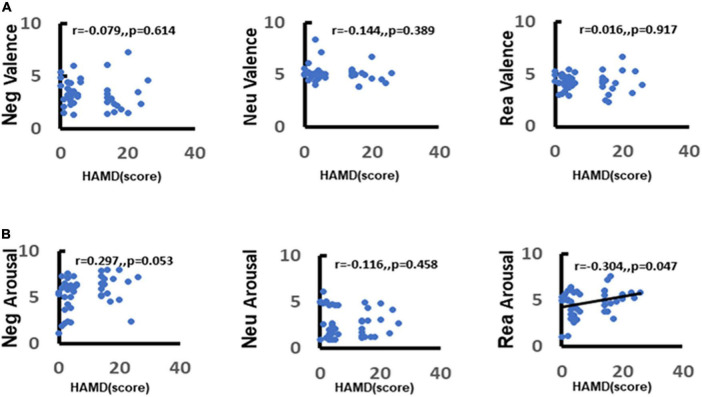
Correlation between depression and emotion regulation. **(A)** Demonstrates the correlation between depression and valence in response to different stimuli (negative, neutral, reappraisal). **(B)** Demonstrates the correlation between depression and arousal in response to different stimuli (negative, neutral, and reappraisal).

To further explore the correlation between depression and emotion regulation, the present study used the Spearman’s correlation between arousal scores of different stimulus pictures and total HAMD-17 items. It was found that there was a significant positive correlation between depression scores and negative stimulus picture arousal scores (*r* = 0.33, *p* = 0.029) (Figure 7A). The more severe the depressive symptoms, the higher the arousal to negative stimulus pictures and this association was not observed between neutral stimulus pictures and reappraisal stimulus pictures (neutral: *r* = −0.13, *p* = 0.4; reappraisal: *r* = 0.19, *p* = 0.23) (Figure 7B).

### Correlation Between Depression and Late Positive Potential Amplitudes

**FIGURE 8:**
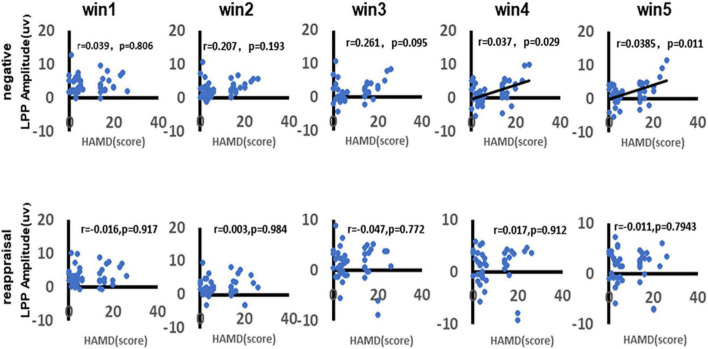
Correlation between depression and LPP amplitudes. As shown in figure, the first row demonstrates the correlation between depression and LPP amplitudes in response to negative stimuli of different time windows (win1, win2, win3, win4, and win5). The second row demonstrates the correlation between depression and LPP amplitudes in response to reappraisal stimuli of different time windows (win1, win2, win3, win4, and win5). Only the significant correlation would show a trend line.

As shown in Figure 8, there was a significant positive correlation between depression and LPP amplitudes in response to negative stimuli during the time win4 (*r* = 0.037, *p* = 0.029) and time win5 (*r* = 0.0385, *p* = 0.011), while there no significant correlation could be found in time win1 (*r* = 0.039, *p* = 0.806), time win2 (*r* = 0.297, *p* = 0.193), and time win3 (*r* = 0.261, *p* = 0.095). As for the LPP amplitudes in response to reappraisal stimuli, there no significant correlation could be found in any time windows (win1: *r* = 0.016, *p* = 0.917; win2: *r* = 0.003, *p* = 0.984; win3: *r* = 0.047, *p* = 0.722; win4: *r* = 0.017, *p* = 0.912; win5: *r* = 0.011, *p* = 0.794).

### Analysis of the Correlation Between Depression and the Neural Oscillations

**FIGURE 9:**
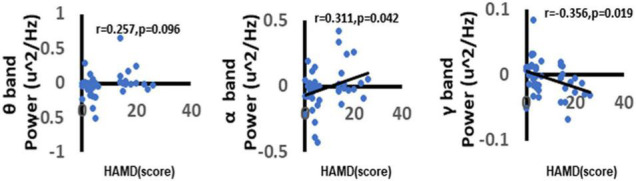
Correlation between depression and neural oscillations. As shown in figure, there was a significant positive correlation between depression and α oscillations. Besides, we can also find that there was a significant negative correlation between depression and γ oscillations. While there no significant correlation between depression and θ oscillations. Only the significant correlation would show a trend line.

To further explore the correlation between depression and the neural oscillations. In the current study, we used the Spearman correlation method to explore the correlation between different frequency power ERSP values and HAMD-17 total score. We found a significant positive correlation between depression scores and alpha-band oscillation (*r* = 0.311, *p* = 0.042). The more severe the depressive symptoms, the higher the alpha-band oscillations in the central area when faced with a negative picture stimulus. There was a significant negative correlation between gamma-band oscillations (*r* = −0.356, *p* = 0.019). The more severe the depressive symptoms, the higher the gamma-band oscillations in the central area when faced with a negative picture stimulus (Figure 9).

### Correlation Analysis Between Depression and Functional Connectivity

**FIGURE 10:**
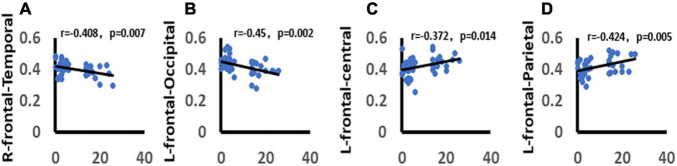
Correlation between depression and functional connectivity. **(A)** Demonstrates the significant negative correlation between depression and right frontal-temporal lobe function connectivity. **(B)** Demonstrates the significant negative correlation between depression and left frontal-occipital lobe function connectivity. **(C)** Demonstrates the significant positive correlation between depression and left frontal-central areas function connectivity. **(D)** Demonstrates the significant positive correlation between depression and right frontal-parietal lobe function connectivity. Only the significant correlation would show a trend line.

We analyzed the correlation between the total score of HAMD-17 and functional connectivity. It was found that there was a significant negative correlation between the total score of HAMD-17 and alpha band wPLI between right frontal and temporal lobes in the win4 time window (*r* = −0.408, *p* = 0.007). There was a significant negative correlation between alpha-band wPLI and the left frontal lobe, occipital lobe in win5 time window (*r* = −0.45, *p* = 0.002). In addition, the HAMD-17 total score was significantly positively correlated with alpha-band wPLI between the left frontal lobe and central areas in the win5 time window (*r* = 0.372, *p* = 0.001), and with wPLI between the right frontal lobe and parietal lobe in the alpha band in win5 time window (*r* = 0.424, *p* = 0.005) (Figure 10).

### Correlation Analysis Between Depression and Network Metrics

**FIGURE 11:**
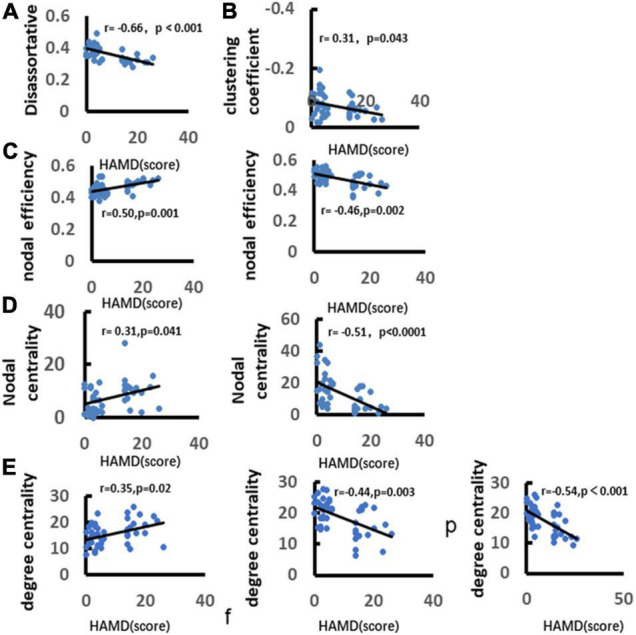
Correlation between depression and alpha band network metrics. **(A)** Demonstrates significant positive correlation between depression and disassortative. **(B)** Illustrates significant negative correlation between depression and clustering coefficient. **(C)** Demonstrates significant positive correlation between depression and nodal efficiency over frontal and parietal lobes. **(D)** Demonstrates significant negative correlation between depression and nodal betweenness centrality. **(E)** Illustrates significant positive correlation between depression and nodal degree centrality. Only the significant correlation would show a trend line.

We analyzed the correlation between depression and graph theory index. It was found that there was a significant negative correlation between depression and the clustering coefficient (*r* = −0.66, *p* < 0.001). There was a significant positive correlation between depression with disassortative (*r* = 0.31, *p* = 0.043). Depression was significantly positively correlated with the node efficiency over frontal lobe (*r* = 0.50, *p* = 0.001), but negatively correlated with the nodal efficiency over parietal lobe (*r* = −0.46, *p* = 0.002). Depression was significantly positively correlated with nodal centrality over frontal lobe (*r* = 0.31, *p* = 0.041), and negatively correlated with nodal centrality over parietal lobe (*r* = −0.51, *p* < 0.0001). In addition, depression was significantly positively correlated with the nodal degree centrality over frontal lobe (*r* = 0.35, *p* = 0.02), and negatively correlated with nodal degree centrality over parietal and occipital lobes (parietal lobe: *r* = −0.44, *p* = 0.003; occipital lobe: *r* = −0.54, *p* < 0.0001) (Figure 11).

## Discussion

### Depression May Affect the Emotional Regulation Ability of Patients With Mild Cognitive Impairment

The study mainly compared the differences in valence and arousal rating scores in the completion of emotion regulation tasks between the two groups with and without depressive symptoms in MCI and found that individuals with MCI combined with depressive symptoms had more significant arousal to pictures of negative stimuli. And the more severe the depressive symptoms, the stronger arousal in response to negative stimuli, which further suggested that depressive symptoms do affect the processing of emotion regulation. Previous studies have demonstrated that depressed individuals have increased attention and memory for negative information and decreased attention and memory for positive information (Erickson et al., 2005; Leyman et al., 2007), showing dominant processing of negative information (Clark et al., 2009). Individuals with depression usually pay more attention to negative stimuli (Peckham et al., 2010). In the cognitive theory of emotional disorders, the schema-based model of cognition posits that depression has a negative bias in all aspects of information processing, such as perception, attention, memory, and reasoning (Phillips et al., 2003a,b) and that a systematic negative bias is a central feature of depression (Ochsner and Gross, 2005). Cognitive theories of depression suggest that this cognitive bias persists throughout the depressive episode and may be necessary for the development, maintenance, and recurrence of depression (Hariri et al., 2003). Research over the past 50 years supports the view that depression and depression risk are characterized by negative biases, usually a lack of positive biases, self-referential processing, interpretation, attention and memory, and maladaptive cognitive emotion regulation strategies. There is also evidence that the lack of cognitive control over emotional consistency materials is the basis of these cognitive processes. Specifically, studies have shown that it is difficult to inhibit and disengage from harmful materials in working memory: (1) increases the use of maladaptive emotion regulation strategies (such as rumination), decreases the use of adaptive emotion regulation strategies (such as reappraisal), and potentially impedes flexible selection and implementation of emotion regulation strategies. (2) It is related to the negative biases in attention. (3) Lead to negative biases in long-term memory. In addition, studies have shown that these cognitive processes exacerbate and sustain the typical negative emotions of depressive episodes (Villalobos et al., 2021).

### Effects of Depressive Symptoms on Late Positive Potential

The study showed that negative stimulus pictures were more likely to provoke higher LPP amplitude values in individuals with MCI comorbid depressive symptoms. The LPP is an ERP component that appears approximately 300 ms after stimulus onset and maintains its extensive supra-scalp distribution during the presentation of the emotional stimulus (Hajcak et al., 2010). Numerous studies have shown that the LPP is enhanced (i.e., more responsive) after the presentation of pleasant and unpleasant stimuli compared to neutral stimuli (Hajcak et al., 2010). The LPP has also been shown to be greater with stimuli with higher arousal levels (Schupp et al., 2004). This approach (i.e., measuring the LPP response to emotional stimulus presentation) is a reliable indicator of emotional arousal (Hajcak and Olvet, 2008). This is more consistent with previous studies that concluded that negative emotions tend to enhance ERP amplitude (Hajcak and Olvet, 2008; Sass et al., 2010) and that negative stimuli are more likely to provoke attention in individuals with noted depression (Sass et al., 2010). This may be because individuals with MCI combined with depressive symptoms show impairments in processing negative stimuli enhancing the allocation of attention to negative emotions in depressed individuals when confronted with these unpleasant stimulus pictures (Hajcak et al., 2010).

### Effect of Depressive Symptoms on Neural Oscillations

In this study, we compared the characteristics of temporal-frequency changes after processing different stimulus pictures in two groups and found that frontal theta and alpha oscillations were significantly higher and gamma oscillations were significantly lower in MCI comorbidly depressed individuals compared to controls in a harmful picture stimulation task. Many studies have found that theta oscillations are associated with working memory, attention, and emotional processing (Knyazev et al., 2009; Itthipuripat et al., 2013). Emotional stimuli induce greater theta event-related synchronization (ERS) in the posterior cortical area (Aftanas et al., 2008) than neutral stimuli, suggesting that theta oscillations modulate the attention elicited. Emotional stimuli automatically capture attention, and the process of negative emotions is associated with increased neural activity in regions associated with the attentional network (Anderson et al., 2003). Thus, the increase in theta oscillations may indicate that MCI depressed individuals require more attentional resources for processing pictures of negative stimuli than the controls group (Wei et al., 2017). Alpha oscillations have been shown to be negatively correlated with brain activity (Fingelkurts et al., 2007), and analyses have shown that subjective reports of emotional arousal are directly related to activation of the cerebral cortex (i.e., a decrease in alpha). Activation of the cerebral cortex is associated with a decrease in the alpha oscillations and increases with increasing arousal of the image, independent of stimulus potency. Asymmetry in alpha activity is associated with individuals’ affective, emotional and depressive states (Cacioppo, 2004). The higher the alpha power, the lower the brain activity (Kline et al., 2007). The increase in low-frequency oscillations (theta and alpha) and the decrease in high-frequency oscillations (gamma) may reflect deficits in cognitive processing. Thus, we can expand our understanding of the characteristics of cognitive processes in depression, and alterations in EEG oscillations can help evaluate the effectiveness of depression treatment or treatment strategies. Suppose the low-frequency high activity and low high-frequency activity of EEG oscillations in an emotion regulation task are alleviated after treatment. In that case, the treatment modality for depression is effective, a hypothesis that needs to be tested in future follow-up studies.

### Effects of Depressive Symptoms on Functional Connectivity

Emotion regulation is advanced processing that is not the result of independent activity in one brain region. The generation, perception and regulation of emotions depending on the synergy of multiple brain regions or networks (Morawetz et al., 2020). Our study found that MCI combined with depressive symptoms showed increased in the left frontal-occipital lobe, right frontal-parietal lobe functional connectivity in response to negative stimuli, The increase in the response indicates that depressed MCI patients needed to consume more neural resources and activate specific neural networks in the process of negative stimuli image processing to achieve a similar level as the control group (Harvey et al., 2005; Fitzgerald et al., 2008). Furthermore, correlation analysis showed that the increased functional connectivity were positive correlated with depressive symptoms, strengthening the argument. While we observed that there was a functional connectivity decrease in left frontal-occipital lobe and right frontal-temporal lobe in patients with depressive symptoms in response to negative stimuli. That is probably because, regarding negative stimulus, MCI depressive individuals showed decreased in the processing efficiency (Ma, 2020), and previous studies have shown that depressed individuals have more pronounced functional connectivity decline (Greco et al., 2021). What’s more, our study also found that depressive symptoms were negative correlated with the decreased functional connectivity, which further strengthened our speculation. This is of great significance for the future study on cognitive and emotional rehabilitation.

### Effects of Depressive Symptoms on Network Metrics

The clustering coefficients of the network quantify the degree of connectivity between adjacent regions and are therefore considered the ability of the network to process local information (Masuda et al., 2018). Node efficiency can measure the efficiency of information exchange in brain regions associated with emotion regulation (Pan et al., 2018). The results of graph theory analysis showed that for the MCI comorbid depressive symptoms group, global network metrics regarding disassortative were significantly decreased, indicating that the connectivity between brain network regions was decreased in MCI comorbid depressive symptoms individuals during the processing of emotional stimuli (Wei et al., 2010). In contrast, nodal and modular network metrics such as clustering coefficients, nodal efficiency, degree centrality, and betweenness centrality significantly increased in the frontal lobe. It decreased in the parieto-occipital lobe, suggesting that MCI comorbid depressive individuals have decreased top-down information processing during the processing of negative stimuli.

Further correlation analysis revealed that the more severe the depressive symptoms, the more significant the increase in nodal efficiency, degree centrality and betweenness centrality values in the frontal lobe, while the more severe the depressive symptoms, the more significant the decrease in nodal efficiency, degree centrality and betweenness centrality values in the parietal-occipital lobe. Previous studies have also shown that frontal and parietal regions play an essential role in emotional processing (Mitchell et al., 2008; Escolano et al., 2011). Depressive symptoms are closely related to functional brain connectivity, and in positive emotion states, the brain is more active, with stronger connections between brain network regions and faster information transfer. However, in negative emotional states, the brain is inactive, there are fewer connections between brain network regions, and the speed of information transfer decreases (Hajcak et al., 2010).

### Summary and Limitations

In this study, by exploring the neural mechanisms underlying the occurrence of impaired emotion regulation in MCI-combined depressed individuals, we found that MCI-combined depressed individuals have a negative preference and dominant processing of negative stimuli, as evidenced by the increased arousal to negative stimuli in MCI-combined depressed individuals, and the ERP findings suggest that the LPP amplitude values to negative stimuli in MCI-combined depressed individuals increased significantly. This further supports the findings of the behavioral study. The results of time-frequency domain analysis showed that theta and alpha oscillations were enhanced, and gamma oscillations were decreased in MCI comorbid depressive individuals in response to harmful stimuli, suggesting that the impairment of emotion regulation in MCI comorbid depressive individuals may be related to the enhancement of low-frequency activity oscillations and the weakening of high-frequency activity oscillations. Functional connectivity analysis suggested that MCI depressed individuals manifested a dysfunction in alpha band connectivity in response to negative stimuli, suggesting that abnormal alpha band network connectivity may be a marker of depressive symptoms. Further correlation analysis showed that the more severe the degree of depressive symptoms, the stronger the arousal of subjects to harmful stimuli, and the higher the LPP amplitude, the stronger the arousal of negative stimuli, further supporting the conclusion that negative preference exists in MCI individuals with combined depressive symptoms. The severity of depressive symptoms was highly correlated with the alpha and gamma oscillations, as well as the alpha band functional connectivity. This suggested that the occurrence of depressive symptoms affects emotional processing. The present study is the first to investigate the neural mechanisms underlying the decreased emotion regulation in MCI depressed individuals, and it has some innovative significance; however, there are still some limitations in this study. First, the sample size was relatively small, with insufficient evidence. Future studies need to expand the sample size and enhance the argumentation. Second, this study is a cross-sectional study, which cannot elucidate the causal relationship between depressive symptoms and cognitive function and emotion regulation. Future studies can use neuromodulation techniques to intervene and further strengthen the argument. Third, the present study only compared the difference in the magnitude of LPP between the two groups. LPP is also applicable to other assessment methods, such as the time it takes to rise from baseline to peak and decline back to baseline can be used as an indicator to characterize emotional responses. According to previous studies, the duration of LPP elicited by emotional stimuli can be adjusted by cognitive guidance. Future studies can further utilize the duration of LPP as an indicator of emotion regulation. Fourth, the present study did not explore the activation of brain regions for emotional picture processing, and in the next stage of the study, we will extract the brain network features related to emotional processing based on the source level to strengthen the argumentation further. Finally, in the current study, negative pictures were employed in the emotion regulation task, and there no significant difference in valence rating scores, while arousal was significantly increased than that used for negative viewing. Although this difference did not affect the main results of the study, but we should control the valence and arousal between negative viewing and reappraisal.

## Conclusion

Our research revealed that MCI depressed individuals exhibit a preference for negative stimuli. Gamma, alpha and theta brain rhythms play essential roles in emotion stimuli processing. MCI depressed individuals showed an increase in theta and alpha oscillations while gamma oscillations decreased. The severity of depressive symptoms was closely associated with changes in the alpha and gamma band oscillations. In addition, when presented with negative stimuli, MCI depressed individuals manifested a dysfunction in alpha band connectivity. Our study suggested that the severity of depressive symptoms highly correlates with the ability to regulate emotion.

Most importantly, MCI in depressed individuals exhibited a higher tendency toward processing negative stimuli. The findings may owe to both increased low-frequency oscillations activity and decreased high-frequency activity, the depressive symptoms aggravates the impairment of alpha-band network connectivity. Therefore, we speculated that these might be important reasons contributing to the impairment of emotion regulation in MCI depressed individuals.

## Data Availability Statement

The raw data supporting the conclusions of this article will be made available by the authors, without undue reservation.

## Ethics Statement

The studies involving human participants were reviewed and approved by the Ethics Committee of Shanghai Tongji Hospital. The patients/participants provided their written informed consent to participate in this study.

## Author Contributions

ML: study design, data collection, statistical analysis, data interpretation, manuscript editing, and literature search. JM: data collection, statistical analysis, and manuscript revision. C-YF: study design, manuscript revision, review, and editing. JY: data collection, manuscript translation, and literature search. S-SX: study design, data collection, EEG data preprocessing, and data analysis. W-XX, R-RL, and WZ: data collection and literature search. Z-MX: data preprocessing and literature search. Y-JL: study design, supervision, and manuscript revision. Y-XL: study design, supervision, manuscript revision, and funding acquisition. All authors contributed to the article and approved the submitted version.

## Conflict of Interest

The authors declare that the research was conducted in the absence of any commercial or financial relationships that could be construed as a potential conflict of interest.

## Publisher’s Note

All claims expressed in this article are solely those of the authors and do not necessarily represent those of their affiliated organizations, or those of the publisher, the editors and the reviewers. Any product that may be evaluated in this article, or claim that may be made by its manufacturer, is not guaranteed or endorsed by the publisher.
